# Resurrected memories: Sleep-dependent memory consolidation saves memories from competition induced by retrieval practice

**DOI:** 10.3758/s13423-021-01953-6

**Published:** 2021-06-25

**Authors:** Xiaonan L. Liu, Charan Ranganath

**Affiliations:** 1 Center for Neuroscience, University of California, Davis, CA 95618 USA; 2 Department of Psychology, University of California, Davis, CA USA

**Keywords:** Retrieval practice, Retrieval-induced facilitation, Memory consolidation, Sleep

## Abstract

Retrieval practice improves retention of tested information, and it can either impair or facilitate retention of untested information. Here, we investigated how semantic relatedness, episodic context, and sleep-dependent memory consolidation determine the effects of retrieval practice on retention of untested items. Participants studied lists of scene-word associations. Each scene was associated with two different words (“pairmates”) that were either semantically related or unrelated and either in the same (temporally close) or different lists (temporally far). In three experiments, retrieval practice of scene-word associations facilitated retention of unpracticed, temporally close pairmates and impaired retention of temporally far, semantically unrelated pairmates. Critically, retrieval practice impaired retention of temporally far, semantically related pairmates if participants were unable to sleep during the retention interval, but it facilitated retention of these items if participants were able to sleep. Our findings suggest that sleep extends the benefits of testing to related information learned in temporally separate episodes.

**Supplementary Information:**

The online version contains supplementary material available at 10.3758/s13423-021-01953-6.

## Introduction

Many models of memory conceptualize remembering as a process that simply involves activation of a stored memory trace. Considerable evidence, however, suggests that episodic memory is more dynamic, such that repeated retrieval of an event (“retrieval practice”) dramatically enhances the ability to retain the practiced information (Karpicke & Roediger, 2008; Roediger & Butler, 2011; Rowland, 2014). Although retrieval practice clearly benefits retention of practiced items, it has more complex effects on information that was not previously retrieved. Many studies have demonstrated that retrieval practice can impair retention of related information that was not previously retrieved (Anderson, 2003; Anderson et al., 1994; Anderson & Hulbert, 2020; Bäuml & Kliegl, 2017; Jonker et al., 2013; Raaijmakers & Jakab, 2013), a phenomenon called *retrieval-induced forgetting* (RIF), whereas other studies have shown that retrieval practice facilitates retention of untested information, a phenomenon called *retrieval-induced facilitation* (Chan, 2009; Chan et al., 2006; Jonker et al., 2018; Rowland & Delosh, 2014).

Why does retrieval sometimes impair and sometimes facilitate retrieval of related information that was not actively retrieved? In general, theories suggest that practicing one item can lead to forgetting of competing items due interference or inhibition (Anderson, 2003; Anderson et al., 1994; Jonker et al., 2013; Lewis-Peacock & Norman, 2014; Newman & Norman, 2010; Raaijmakers & Jakab, 2013), but this effect can be overcome if subjects can intentionally interrelate the items (Anderson & McCulloch, 1999; Chan, 2009; Goodmon & Anderson, 2011). For example, Anderson et al. (1994) showed that retrieving a target item impairs memory of related “non-targets” that were not explicitly retrieved, leading to RIF. Moreover, Chan (2009) demonstrated that instructing participants to integrate information learned from each sentence of an article and relate them to each other during encoding can resolve competition and lead to facilitation. At a computational level, the dynamic between RIF and facilitation can be explained by the *non-monotonic plasticity hypothesis* (Lewis-Peacock & Norman, 2014; Newman & Norman, 2010; Ritvo et al., 2019). This model proposes that retrieval practice strongly co-activates, and thereby strengthens, representations of non-targets that are integrated with the targets, whereas non-targets that are not integrated with the targets are only moderately activated, leading to weakening of non-target representations.

Temporal context might also play a role in moderating effects of retrieval practice. Several findings suggest that episodic memory is temporally organized, such that retrieval of one item facilitates recall of other items that were studied in close temporal proximity (Howard & Kahana, 2002). Accordingly, we might expect the benefits of retrieval practice to spill over onto other temporally proximal items. Consistent with this idea, available evidence suggests that retrieval can facilitate retention of untested items from the same episodic context (Jonker et al., 2018; Rowland & Delosh, 2014).

Here, we considered another possibility – that the fate of untested items might be determined by sleep-dependent memory consolidation. Consistent with this account, in Chan (2009), a 24-h delay eliminated the RIF shown with a 20-min delay in the “low-integration” condition and led to retrieval-induced facilitation in the “high-integration” condition. Lewis and Durrant (2011) highlighted evidence suggesting that memories may be reactivated during sleep, and they proposed that repeated reactivation of memories in different combinations strengthens shared elements and facilitates the formation of schematic representations of the relationships between stimuli (see also Tononi & Cirelli, 2014). Based on these ideas, we investigated whether sleep-dependent memory consolidation could mitigate the competition that leads to impairment and instead facilitate retention of non-tested items.

**Fig. 1:**
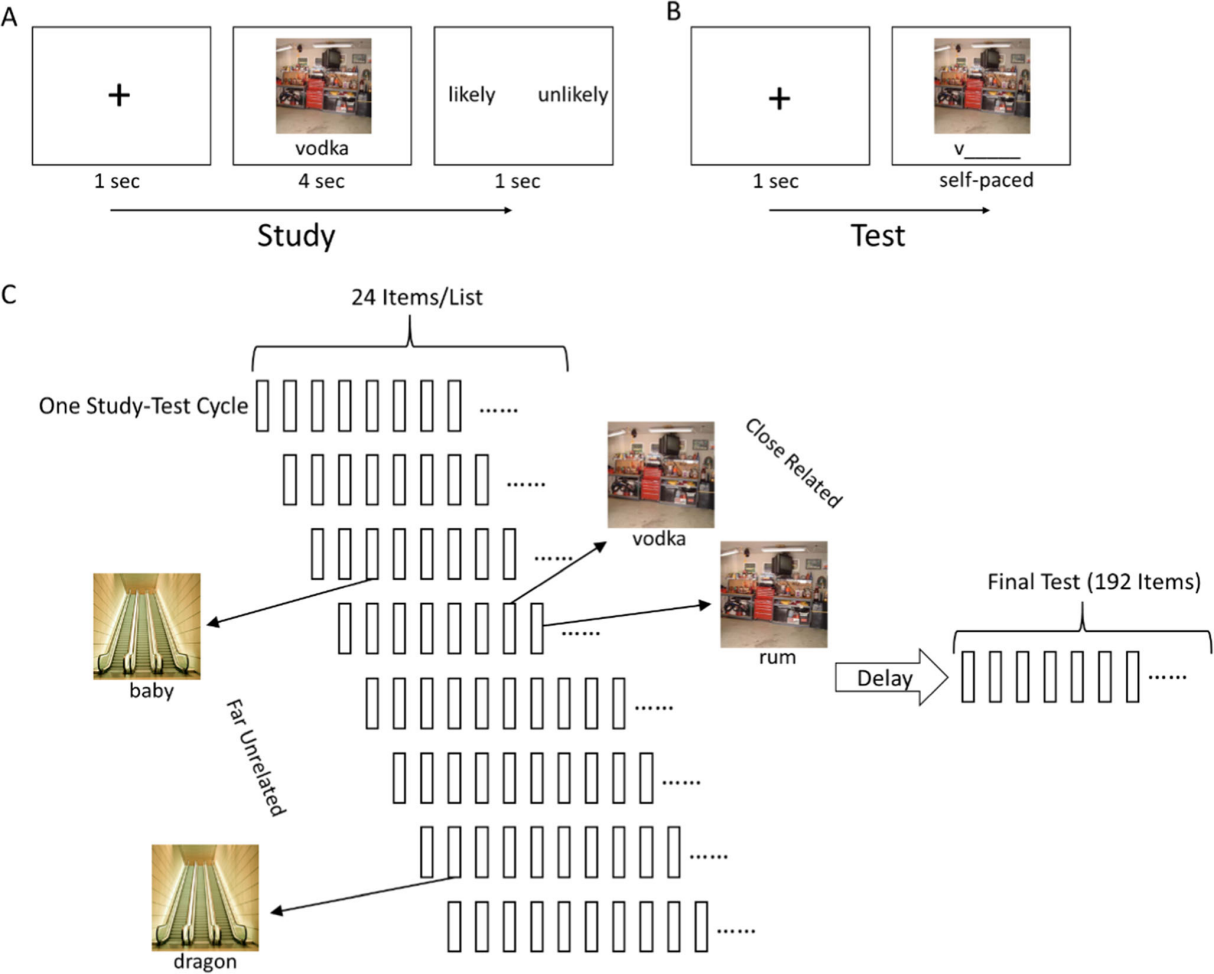
(**A**) Graphic representation of the study procedure. (**B**) Graphic representation of the test procedure during retrieval practice and the final test. (**C**) Graphic representation of the overall experimental paradigm. The delay was manipulated between-subject as short (10 min) vs. long (24 h) in Experiment 1 and as wake vs. sleep in Experiment 2 and Experiment 3

Here, we report results from three experiments testing the effects of semantic relatedness, episodic context, and sleep on retrieval-induced effects on untested information. We adapted a paradigm introduced by Jonker et al. (2018) to test the effects of retrieval practice on retention of arbitrary scene-word associations (Fig. 1). In Experiment 1, we manipulated the extent to which retrieved and non-retrieved items were semantically and temporally related, and we compared retention of these items between subjects who were tested immediately and subjects who were tested after a 1-day delay. Experiments 2 and 3 used a similar design, except that the retention delay was held constant, and we instead manipulated whether the delay included a night of sleep.

## Experiment 1

### Method

#### Participants

Seventy-eight students (50 participants identified as female and 28 participants identified as male) from the University of California, Davis, participated in exchange for partial course credit. All reported fluency in English and normal or corrected-to-normal vision. Participants were randomly assigned to two groups (short delay vs. long delay) with 39 participants in each group. Four participants in the short-delay group and two participants in the long-delay group were excluded due to low accuracy during retrieval practice (below three standard deviations (SDs) from the mean). Our sample size was determined using unpublished work examining retrieval-induced facilitation from our laboratory with an a priori power analysis by GPower (Faul et al., 2009) with power (1-β) set at 0.80 and α = 0.05. Prior data from our laboratory showed retrieval-induced facilitation with a medium effect size (Cohen’d=.5, Cohen, 1992), which requires at least 34 participants to detect.

#### Materials

Ninety-six scene images were selected from Konkle et al. (2010). We selected 48 pairs of semantically related concrete nouns (mean semantic feature overlap = .56) and 96 concrete nouns without feature overlap with any other words from English Semantic Word-Pair Norms (Buchanan et al., 2013). For each participant, 96 unrelated words were randomly grouped into 48 unrelated pairs and each scene was randomly associated with two words or “pairmates” in either a related word pair or an unrelated word pair with the restriction that no pairing had a strong pre-existing contextual association (e.g., kitchen scene paired with the word “blender”), resulting in 192 scene-word associations and 96 groups of pairmates sharing the same scene. The number of trials in each condition is presented in Table 1.

**Table 1:** The number of trials in each condition in three experiments

Conditions		Related			Unrelated		
		Control	Non-target	Target	Control	Non-target	Target
Experiment 1	Adjacent	16	16	16	16	16	16
	Close	8	8	8	8	8	8
	Far	8	8	8	8	8	8
Experiment 2/3	Adjacent	16	16	16	16	16	16
	Far	16	16	16	16	16	16

#### Design

The experimental design was adapted from a paradigm introduced by Jonker et al. (2018), in which we investigated retrieval-induced facilitation for scene-item associations. In this study, participants performed repeated study-test cycles for each list, and then retention of these associations was assessed on a final test. The factorial experimental design incorporated three within-subject factors – retrieval practice, temporal distance, and semantic relatedness – and one between-subject factor – the delay between retrieval practice and final test. As described in more detail below, participants studied eight lists of scene-word associations. The retrieval practice manipulation resulted in three types of trials: For some the scene-word associations, one pairmate, the retrieval “*target*,”^1^ was repeatedly tested after study. We refer to the non-practiced pairmate as a “*non-target.*” Finally, for “*control*” associations, neither of the pairmates were practiced.

The temporal distance manipulation in Experiment 1 focused on the distance between the practiced scene-item association and the unpracticed pairmate. Unpracticed pairmates were either “*adjacent*” (i.e., the two associations were presented successively during study), “*close*” (i.e., the two associations were within the same list but with at least one association from another group in between), and “*far*” (i.e., the two associations were studied in different lists with four intervening lists in between). With this design, adjacent and close pairmates were associated with similar temporal contexts, whereas far pairmates were associated with very different contexts. In the *far* condition, the non-target was always studied in an earlier list than the target, in order to ensure that participants learned both associations before retrieval practice. Finally, pairmates were either semantically *related* or *unrelated*.

To investigate the effects of memory consolidation in this experiment, participants were randomly assigned to one of two groups – one group completed a final test on all of the learned associations 10 min after the last study delay and 24-h delay. Thus, participants in the 24-h delay condition had the opportunity to sleep between study and test.

#### Procedure

In each list, participants first studied 24 scene-word associations. As shown in Fig. 1, each study trial began with a fixation cross for a period of 1 s. Each association was presented for 4 s and participants were instructed to remember the association and indicate with a key press whether the object, which the word referred to, was likely to be seen in the scene within 1 s after the presentation of scene-word associations. After initial study of all associations for a given list, participants were given a practice test of retrieval targets in this list assigned previously. Each retrieval trial involved the scene plus a one-letter word stem, and participants were to type in the whole target word. No feedback was given after each retrieval practice trial. After each list, participants were given a short self-paced break before moving on to the next list. Participants cycled through the eight lists twice to ensure strong encoding. The order of trials within each list was re-randomized in the second cycle.

Participants were asked to play Sudoku with pencil and paper for 10 min after the two cycles of encoding and retrieval practice. For the short-delay group, an unexpected final test was given immediately after the 10-min delay. For the long-delay group, participants were asked to return the next day and the final test was given during the second visit. During the final test, participants were shown a scene along with a one-letter word stem and prompted to type in the correct word. The test was self-paced. In order to prevent any output interference (Anderson et al., 1994), the order of all non-practiced associations and retrieval targets were separately randomized and retrieval targets were tested after all non-practiced associations. Moreover, for control trials, only the first tested pairmate associated with each scene was included in the analyses.

### Results

On average, during retrieval practice, subjects correctly recalled 74.4% (SD = .21) trials in the first round and 85.7% (SD = .20) trials in the second round. Table 2 presents the means and standard deviations for final test accuracy in different conditions.

**Table 2:** Final recall accuracy (mean percent correct) for Control, Non-target and Target trials, and accuracy difference between Non-target and control trials as a function of temporal distance, semantic relatedness and delay in Experiment 1

Conditions		Short Delay				Long Delay			
		Control	Non-target	Target	Non-target > Control	Control	Non-target	Target	Non-target > Control
Unrelated	Adjacent	.62(.21)	.67(.24)	.83(.17)	.05(.14)	.31(.23)	.40(.22)	.63(.27)	.09(.15)
	Close	.60(.28)	.70(.21)	.82(.19)	.10(.20)	.34(.24)	.40(.26)	.57(.29)	.06(.17)
	Far	.67(.23)	.60(.21)	.84(.19)	-.07(.19)	.40(.25)	.31(.23)	.62(.26)	-.09(.18)
Related	Adjacent	.75(.22)	.80(.17)	.91(.13)	.05(.17)	.47(.27)	.55(.26)	.70(.27)	.09(.16)
	Close	.73(.23)	.81(.20)	.94(.09)	.07(.19)	.52(.28)	.60(.32)	.72(.28)	.08(.22)
	Far	.80(.17)	.69(.21)	.91(.11)	-.11(.18)	.53(.29)	.62(.27)	.74(.27)	.08(.19)

Standard deviations are shown in parentheses

#### Effects of retrieval practice on retention of non-targets

To examine retrieval-induced facilitation and competition, our analyses focused on recall of non-target and control items on the final test. A 2 (Trial Type: non-target vs. control) × 3 (Temporal Distance: adjacent, close, far) × 2 (Semantic Relatedness) × 2 (Delay) mixed ANOVA revealed a four-way interaction, *F*(2,140) = 3.78, *p* = .025, *η*_*p*_^*2*^ = .051. These findings indicate that the degree to which retrieval facilitated or impaired retention of non-targets varied according to Semantic Relatedness, Temporal Distance, and the retention interval. To break down this effect, we separately examined the data at three different levels of Temporal Distance.

##### Regardless of delay, retrieval facilitated retention for temporally adjacent and close non-targets

**Fig. 2:**
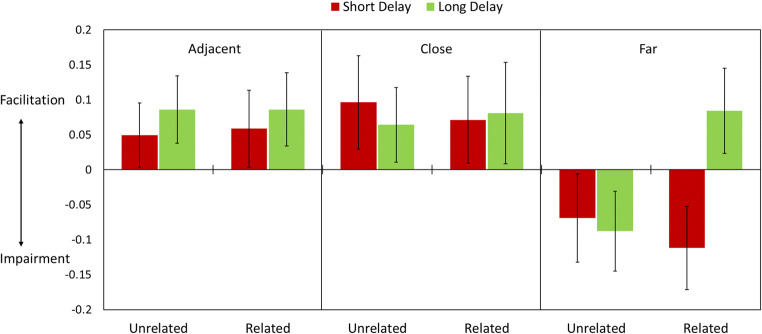
Facilitation and impairment effects in Experiment 1. Graph shows mean final test recall differences between Non-target and Control trials separately as a function of temporal proximity and semantic relatedness. Error bars denote 95% confidence intervals

As shown in Fig. 2, for temporally adjacent and close trials, there were main effects of Trial type (adjacent: *F*(1,70) = 40.86, *p* < .001, *η*_*p*_^*2*^ = .37; close: *F*(1,70) = 23.25, *p* < .001, *η*_*p*_^*2*^ = .25), such that accuracy for non-targets was higher than for control trials, and main effects of Semantic Relatedness (adjacent: *F*(1,70) = 115.08, *p* < .001, *η*_*p*_^*2*^ = .62; close: *F*(1,70) = 47.01, *p* < .001, *η*_*p*_^*2*^ = .40), such that accuracy was generally higher for related trials than for unrelated trials. There were no other significant main effects or interactions (p-values > .1).

##### Regardless of delay, retrieval practice impaired retention of temporally far, semantically unrelated non-targets

For temporally far trials, there was a significant three-way interaction between Trial Types, Semantic Relatedness, and Group (*F*(1,70) = 14.91, *p* < .001, *η*_*p*_^*2*^ = .18). Follow-up analyses of temporally far and unrelated trials revealed that, retrieval impaired temporally far non-targets that were unrelated to targets (main effect of Trial Types for temporally far and unrelated trials: *F*(1,70) = 13.12, *p* = .001, *η*_*p*_^*2*^ = .16). There was no significant interaction between Semantic Relatedness and Group (*F*(1,70) = 0.19, *p* = .66, *η*_*p*_^*2*^ = .003).

##### When tested immediately, retrieval impaired recall of temporally far and semantically related non-targets, but retrieval facilitated retention of these items after a 24-hour delay

Surprisingly, for temporally far and related items, there was a significant interaction between Group and Semantic Relatedness (*F*(1,70) = 20.40, *p* < .001, *η*_*p*_^*2*^ = .23), such that retrieval practice impaired retention for this type of trials in the short-delay (no sleep) group (*F*(1,34) = 13.59, *p* = .001, *η*_*p*_^*2*^ = .29), but facilitated retention in the long-delay (with sleep) group (*F*(1,36) = 7.41, *p* = .010, *η*_*p*_^*2*^ = .17).

Overall, the results showed that: (1) retrieval practice facilitates retention of items sharing a similar temporal context but impairs retention of items learned in a different temporal context, and (2) competition between semantically related, temporally far items switches to facilitation after a long delay with intervening sleep.

## Experiment 2

Experiment 1 provided evidence that retrieval generally enhances retention of pairmates from a similar temporal context, and that, even when pairmates are far apart, there is a surprising delay-dependent switch between retrieval-induced forgetting and facilitation for semantically related information. The latter effect is consistent with the hypothesis that memory consolidation can strengthen associations between memories with shared elements (e.g., Lewis & Durrant, 2011). In Experiment 1, the sleep and no-sleep groups were tested at similar times of day, but the retention interval varied. In Experiment 2, we sought to examine whether sleep could rescue untested items from competition even if the retention interval was held constant. To test this prediction, we tested two groups with a fixed 12-h delay between study and test, but the timing of the sessions was arranged so that one group was awake during the retention interval, and the other group was able to sleep during the retention interval. Experiment 3 was a pre-registered replication of Experiment 2, using identical materials, design, and procedure (https://osf.io/8nzgb).

### Method

#### Participants

Ninety-six students (71 participants identified as female, 24 participants identified as male and one participant selected *Other*, Experiment 2) and 200 students (142 participants identified as female, 53 participants identified as male, and five participants selected *Other*, Experiment 3) from the University of California, Davis participated in exchange for partial course credit. All reported fluency in English and normal or corrected-to-normal vision. All participants were randomly assigned to either a “sleep” or a “wake” group (n = 48 participants/group in Experiment 2; n = 100 participants/group in Experiment 3). In Experiment 2, four participants in the sleep group and three participants in the wake group were excluded due to low accuracy during retrieval practice (below three SDs from the mean) and seven participants in the wake group were excluded due to taking naps between two sessions. In Experiment 3, nine participants in the sleep group and seven participants in the wake group were excluded due to low accuracy during retrieval practice and nine participants in the wake group were excluded due to taking naps between two sessions.

Because Experiment 3 was designed as a replication of Experiment 2, the sample size for this study was determined using the smallest effect size observed in Experiment 2 (*d* = .32) with an a priori power analysis by GPower (Faul et al., 2009) with power (1-β) set at 0.80 and α = 0.05. The analysis showed that this effect requires at least 79 participants to detect. Because, in Experiment 2, approximately 20% of participants were excluded in the wake group, in Experiment 3, we planned to run 100 participants in each group to ensure at least 79 participants in each group would be included in the analyses. Both Experiment 2 and Experiment 3 were conducted online for the ease of scheduling the 12-h delay.

Participants reported no history of neurological or psychiatric disorders, other major medical issues, or use of medication known to interfere with sleep. Participants also reported having a regular sleep the night before the study and between the two sessions (sleep group), which was defined as going to bed no later than 2 am, waking up no later than 10 am, and getting at least 7 h of total sleep.

#### Materials, design, and procedure

The materials and procedure used were identical to Experiment 1 except for the following changes. In Experiment 1, retrieval-induced facilitation was observed in both the adjacent and the close conditions. To simplify the design, in Experiment 2 and Experiment 3, only two levels of temporal distance were included: adjacent and far. The number of trials in each condition is presented in Table 1. Participants in the wake group were asked to finish the first session between 8 am and 12 pm and participants in the sleep group were asked to finish the first session between 8 pm ando 12 am. For both groups, after the first session, participants were instructed to wait 12 h before finishing the second session. At the beginning and end of each session, the Stanford Sleepiness Scale (Hoddes & Dement, 1972), which assesses state sleepiness/alertness on a scale of 1 (extremely alert) to 7 (very sleepy), was completed. An intervening activity survey was given at the beginning of the second session to screen out participants who took naps (wake group) or did not have sufficient sleep (sleep group) between the two sessions.

### Results

#### Vigilance

Stanford sleepiness scores did not differ between sleep and wake groups in Session 1 (Exp. 2: sleep mean = 2.32, wake mean = 2.41, *t* = .43, *p* = .67; Exp. 3: sleep mean = 2.53, wake mean = 2.37, *t* = 1.17, *p* = .24), or in Session 2 (sleep mean = 2.47, wake mean = 2.67, *t* = .97, *p* = .34; Exp. 3: sleep mean = 2.38, wake mean = 2.42, *t* = .31, *p* = .76), suggesting that there were sleepiness differences between groups due to time of day.

In Experiment 2, during retrieval practice, subjects correctly recalled 75% (SD = .18) of trials in the first round and 85.4% (SD = .15) of trials in the second round. In Experiment 3, subjects correctly recalled 73% (SD = .20) of trials in the first round and 84% (SD = .19) of trials in the second round.

Tables 3 and 4 present the means and standard deviations for final test accuracy in different conditions in Experiment 2 and Experiment 3.

**Table 3:** Final recall accuracy (mean percent correct) for Control, Non-target and Target trials, and accuracy difference between Non-target and control trials as a function of temporal distance, semantic relatedness and delay in Experiment 2

Conditions		Wake				Sleep			
		Control	Non-target	Target	Non-target > Control	Control	Non-target	Target	Non-target > Control
Unrelated	Adjacent	.32(.25)	.38(.20)	.53(.25)	.06(.17)	.39(.26)	.44(.21)	.63(.22)	.05(.18)
	Far	.33(.26)	.26(.20)	.61(.25)	-.07(.20)	.43(.26)	.29(.21)	.67(21)	-.14(.15)
Related	Adjacent	.39(.25)	.59(.25)	.75(.20)	.20(.16)	.46(.26)	.61(.22)	.80(.16)	.15(.16)
	Far	.55(.17)	.50(.21)	.74(.22)	-.05(.17)	.55(.25)	.61(.24)	.76(.23)	.06(.18)

Standard deviations are shown in parentheses.

#### Effects of retrieval practice on retention of non-targets

**Fig. 3:**
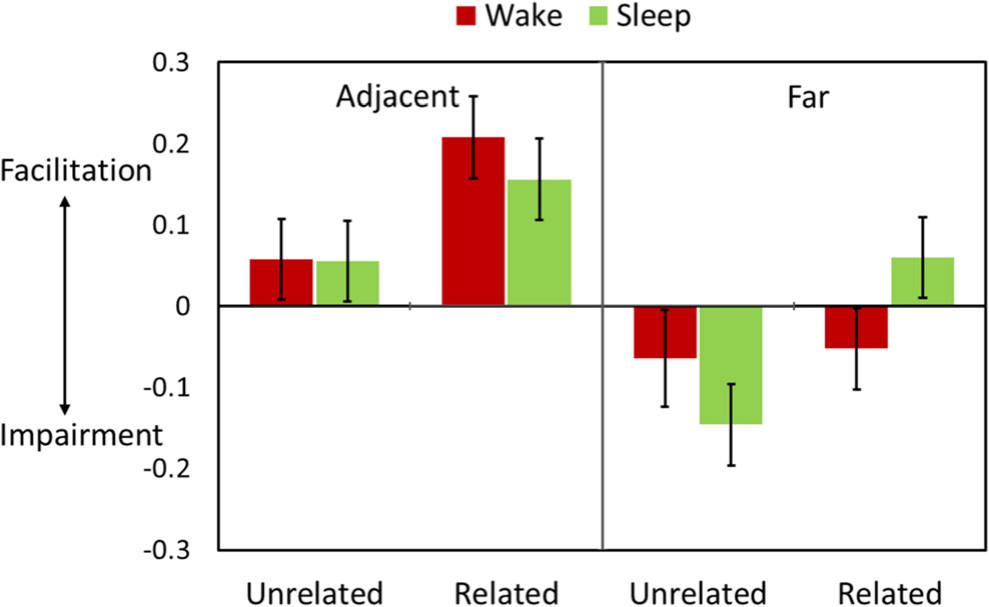
Facilitation and impairment effects in Experiment 2. The graph shows the mean final test-recall differences between Non-target and Control trials separately as a function of temporal proximity (Close vs. Far) and semantic relatedness. Error bars denote 95% confidence intervals

**Fig. 4:**
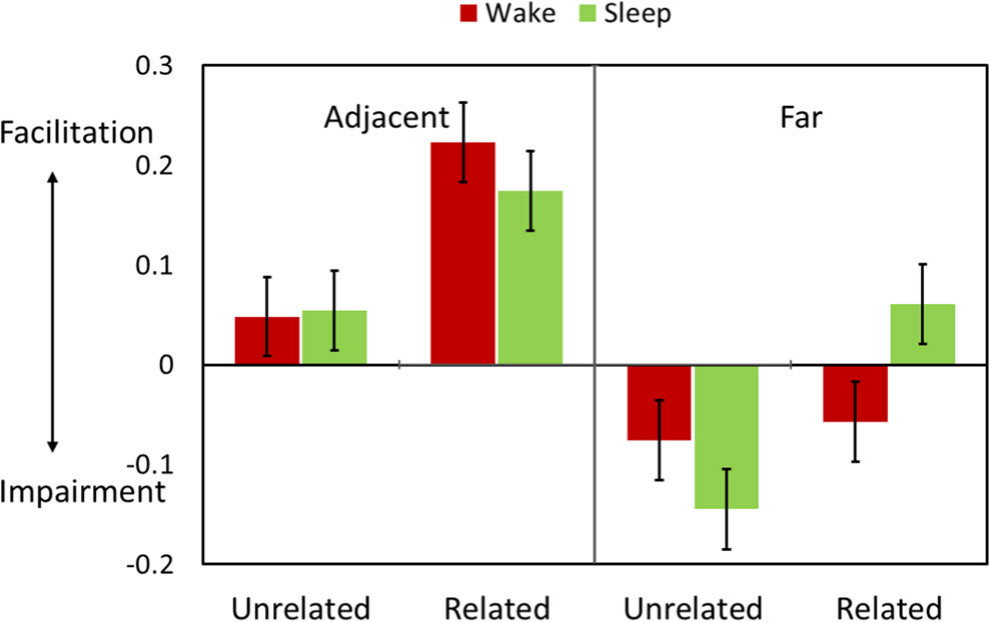
Facilitation and impairment effects in Experiment 3. The graph shows the mean final test-recall differences between Non-target and Control trials separately as a function of temporal proximity (Close vs. Far) and semantic relatedness. Error bars denote 95% confidence intervals

As in Experiment 1, our primary analyses focused on recall accuracy for the non-target and control trials on the final test. A 2 (Trial Type: non-target vs. control) × 2 (Temporal Distance: adjacent, far) × 2 (Semantic Relatedness) × 2 (Group) mixed ANOVA revealed four-way interactions in both Experiment 2 and Experiment 3 (Exp. 2: *F*(1,79) = 10.89, *p* = .001, *η*_*p*_^*2*^ = .12; Exp. 3: *F*(1,173) = 22.28, *p* < .001, *η*_*p*_^*2*^ = .11). These findings confirm that, as in Experiment 1, the effects of retrieval practice on retention of non-targets varied as a function of Retention Interval, Temporal Distance, and Semantic Relatedness. Figures 3 and 4 show that these effects closely parallel what was observed in the immediate- and delayed-recall groups in Experiment 1. To break down this effect, we separately examined the data for temporally adjacent and far trials.

**Table 4:** Final recall accuracy (mean percent correct) for Control, Non-target and Target trials, and accuracy difference between Non-target and control trials as a function of temporal distance, semantic relatedness and delay in Experiment 3

Conditions		Wake				Sleep			
		Control	Non-target	Target	Non-target > Control	Control	Non-target	Target	Non-target > Control
Unrelated	Adjacent	.31(.25)	.36(.20)	.52(.25)	.05(.19)	.35(.26)	.41(.23)	.58(.28)	.06(.17)
	Far	.34(.26)	.27(.20)	.59(.25)	-.07(.19)	.44(.28)	.30(.23)	.65(.25)	-.14(.19)
Related	Adjacent	.35(.27)	.58(.25)	.72(.20)	.23(.21)	.42(.30)	.60(.28)	.74(.27)	.18(.19)
	Far	.56(.25)	.51(.23)	.71(.22)	-.05(.20)	.55(.28)	.61(.20)	.73(.28)	.06(.22)

Standard deviations are shown in parentheses.

##### Regardless of sleep, retrieval practice facilitated retention for temporally adjacent non-targets

As shown in Figs. 3 and 4, for temporally adjacent trials, there were main effects of Trial Type (Exp. 2: *F*(1,79) = 71.36, *p* < .001, *η*_*p*_^*2*^ = .48; Exp. 3: *F*(1,173) = 133.85, *p* < .001, *η*_*p*_^*2*^ = .44), such that accuracy for non-targets was better than for control trials, and main effects of Semantic Relatedness (Exp. 2: *F*(1,79) = 50.86, *p* < .001, *η*_*p*_^*2*^ = .39; Exp. 3: *F*(1,173) = 144.19, *p* < .001, *η*_*p*_^*2*^ = .46), such that performance for related trials were generally better than for unrelated trials. There were also interactions between Trial Type and Relatedness (Exp. 2: *F*(1,79) = 26.24, *p* < .001, *η*_*p*_^*2*^ = .25; Exp. 3: *F*(1,173) = 62.28, *p* < .001, *η*_*p*_^*2*^ = .27), such that the facilitation effect was larger for related items than for unrelated items. There were no other significant main effects or interactions (p-values > .1).

##### Regardless of sleep, retrieval practice impaired recall of temporally far and unrelated non-targets

For temporally far trials, there was a significant three-way interaction between Trial Types, Semantic Relatedness, and Group (Exp. 2: *F*(1,79) = 11.60, *p* = .001, *η*_*p*_^*2*^ = .13; Exp. 3: *F*(1,173) = 20.83, *p* < .001, *η*_*p*_^*2*^ = .11). Follow-up analyses of temporally far and unrelated trials revealed that retrieval impaired temporally far non-targets that were unrelated with targets (main effect of Trial Type: Exp. 2: *F*(1,79) = 27.59, *p* < .001, *η*_*p*_^*2*^ = .26; Exp. 3: *F*(1,173) = 57.80, *p* < .001, *η*_*p*_^*2*^ = .25) and the impairment effect was stronger for the sleep group than for the wake group (interaction between Trial Type and Group: Exp. 2: *F*(1,79) = 4.20, *p* = .044, *η*_*p*_^*2*^ = .05; Exp. 3: *F*(1,173) = 5.65, *p* = .019, *η*_*p*_^*2*^ = .032).

##### Without sleep, retrieval practice impaired recall of temporally far and related non-targets but retrieval practice facilitated retention of these items after post-learning sleep

Surprisingly, for temporally far and related items, there was a significant interaction between Group and Semantic Relatedness (Exp. 2: *F*(1,79) = 8.35, *p* = .005, *η*_*p*_^*2*^ = .096; Exp. 3: *F*(1,173) = 14.00, *p* < .001, *η*_*p*_^*2*^ = .075), such that retrieval practice impaired retention for this type of trial in the wake group (Exp. 2: *F*(1,36) = 3.50, *p* = .069, *η*_*p*_^2^ = .089; Exp. 3: F(1,83) = 6.84, *p* = .011, *η*_*p*_^*2*^ = .076), but facilitated retention in the sleep group (Exp. 2: *F*(1,43) = 5.01, *p* = .030, *η*_*p*_^*2*^ = .10); Exp. 3: *F*(1,90) = 7.24, *p* = .008, *η*_*p*_^*2*^ = .074).

##### Across-study comparison

Experiment 1 had a long retention interval, whereas Experiments 2 and 3 had a shorter retention interval. An exploratory analysis comparing the effects of sleep on semantically related items in the far condition between experiments revealed no significant interaction between Group (sleep vs. no sleep) and Experiment (Exp. 1 vs. Exp. 2 or Exp. 1 vs. Exp. 3) on the magnitude of retrieval-induced facilitation/forgetting (Exp. 1 vs. Exp. 2: *F*(1,149) = 2.11, *p* = .148, *η*_*p*_^*2*^ = .014; Exp. 1 vs. Exp. 3: *F*(1,243) = 1.89, *p* = .171, *η*_*p*_^*2*^ = .008). Thus, there is no evidence to suggest that the effects of sleep in Experiments 1–3 were moderated by retention interval.

In summary, results from the comparison of the sleep and wake group mirrored the differences between the short-delay and long-delay groups seen in Experiment 1, suggesting the delay-dependent switch between facilitation and competition is, in fact, sleep dependent.

## General discussion

The goal of this study was to understand why retrieving a past event sometimes enhances and sometimes impairs retention of related information. The results reaffirm that the benefits of testing generalize beyond the target information that is tested, and that testing improves subsequent retention of non-tested information learned in the same temporal context (e.g., Jonker et al., 2018; Rowland & Delosh, 2014). Moreover, although our paradigm differed from traditional approaches to studying RIF (Anderson et al., 1994), we found that retrieval practice can impair retention of competing information that is not episodically or semantically related to the retrieved item (Anderson & McCulloch, 1999; Chan, 2009; Goodmon & Anderson, 2011). Most importantly, our results show that the same conditions that lead to impairment can lead to facilitation following sleep-dependent memory consolidation. These results suggest that sleep-dependent memory consolidation broadens the benefits of retrieval practice by reactivating semantically related information from temporally separate events.

Previous work has shown that repeated memory retrieval can suppress or weaken representations of semantically related items, leading to RIF (Anderson, 2003; Anderson et al., 1994; Anderson & Hulbert, 2020; Bäuml & Kliegl, 2017; Lewis-Peacock & Norman, 2014; Newman & Norman, 2010), but this effect can be reversed if participants strategically interrelate the retrieval target and competitor (Anderson & McCulloch, 1999; Chan, 2009). We found that episodic context can also lead to facilitation, such that retrieval practice facilitated retention of temporally proximal pairmates, even if participants were not instructed to interrelate them with retrieval targets. The idea that episodic context can promote retention of items that might otherwise compete with one another is exactly what would be expected from context-based models of episodic memory, which suggests that recall of an item can drive reactivation of other temporally proximal items from the same event (Davelaar et al., 2005; Estes, 1955; Howard & Kahana, 2002).

The most surprising results of our studies concern the effects of retrieval on related untested items that were studied far apart in time. Experiment 1 showed that retrieval practice impaired retention of temporally distant, semantically related non-targets at an immediate test, but paradoxically, it facilitated retention of these items at a 1-day delay. In Experiments 2 and 3, the interval between retrieval practice and the final test was held constant, and we instead manipulated whether participants had the opportunity to sleep during the retention interval. Again, results showed that retrieval practice impaired retention of temporally distant, semantically related pairmates, but it facilitated retention of these items for participants who were able to sleep during the retention interval. The common element across all three studies is that sleep rescued, and even strengthened, memories that would have otherwise suffered from competition with practiced items.

Prior studies have shown that sleep may reduce the testing effect (Abel et al., 2019; Bäuml et al., 2014), so one might have expected that sleep would simply attenuate effects of retrieval practice on untested items. Biologically based theories of memory consolidation, however, suggest that sleep might have more complex effects on retention of past experiences. Episodic memory depends on interactions between the hippocampus and neocortex, and considerable evidence suggests that cortico-hippocampal interactions may occur during slow-wave sleep (Diekelmann et al., 2011; Mitra et al., 2016; Oudiette & Paller, 2013; Peigneux et al., 2004).

That said, it is not the case that sleep always produces measurable effects on memory performance. Behavioral effects of sleep-mediated consolidation have been inconsistent across studies and paradigm dependent (Cordi & Rasch, 2021). For example, some studies showed that sleep could protect associative memories against interference (Ellenbogen et al., 2006, 2009), but two recent studies failed to replicate this finding (Bailes et al., 2020; Pöhlchen, 2021). Rather than strengthening all memories or slowing forgetting, it is more likely that the sleep has more selective effects. For example, some models propose that reactivation of memories during sleep may strengthen memories with overlapping or related elements (for reviews, see Lewis & Durrant, 2011; Tononi & Cirelli, 2014). Consistent with these views, empirical studies have found that sleep improved memory for shared properties of newly learned semantic categories (Schapiro et al., 2017) and facilitated incorporation of new information into existing semantic knowledge (Tamminen et al., 2013).

Although current models do not directly address how sleep moderates the effect of retrieval practice on memory, they help to explain why sleep selectively facilitated retention of semantically related pairmates learned in different temporal contexts. Temporal contiguity is sufficient to support retrieval-induced facilitation for near pairmates, but the same factors may inhibit facilitation for far pairmates. It is possible that reactivation during sleep is not gated by temporal context, such that reactivation during sleep is driven by semantic associations. If so, then reactivation of strong memories for tested items might drive activation and strengthening of semantically related pairmates during sleep. In other words, sleep might extend the reach of retrieval practice by allowing the brain to discover links between temporally distant experiences.

It is worth noting that, as in other sleep studies, encoding and retrieval were done at different times of day for the sleep and the wake groups in Experiments 2 and 3. Several factors suggest that it is unlikely that the sleep effect was driven by time of day. First, participants in two groups did not differ in sleepiness/alertness in either the first session or the second session. Second, time of day confounds (e.g., circadian fluctuations in vigilance) would be expected to have a global effect on memory for both control items and for non-targets, and including the control trials in the analyses controlled the global effect. Third, we examined the 16 participants in the wake group who took naps between two sessions. The results suggest that taking naps during the daytime has a similar effect as night-time sleep (results in Online Supplementary Material). That said, it is impossible to completely rule out time of day effects for overnight sleep studies without introducing other potential variables.

In summary, the finding that episodic associations (i.e., through shared temporal context) can lead to retrieval-induced facilitation, rather than competition, is compatible with existing theories of RIF and retrieval-induced facilitation (Anderson, 2003; Anderson & Hulbert, 2020; Bäuml & Kliegl, 2017; Chan, 2009; Lewis-Peacock & Norman, 2014; Newman & Norman, 2010; Ritvo et al., 2019). Our findings add to this picture by suggesting that sleep can significantly extend the benefits of retrieval practice, allowing us to overcome the competitive consequences of memory so that we can pull out the common structure across temporally separated events.

## Supplementary Information

ESM 1 (DOCX 14 kb)

## Footnotes

1 Targets are often referred to as RP+ and non-targets are usually referred to as RP− trials in traditional RIF paradigms*.*
