# Supplementary material for: Resurrected memories: Sleep-dependent memory consolidation saves memories from competition induced by retrieval practice
Source: Psychon Bull Rev. 2021 Jun 25;28(6):2035–44. doi: 10.3758/s13423-021-01953-6 (PMC8642353; doi:10.3758/s13423-021-01953-6)
Supplement: Supplementary file 1 — (DOCX 14 kb) [file 13423_2021_1953_MOESM1_ESM.docx]

**Supplementary Results**

Across Experiments 2 and 3, 16 participants in the Wake groups were excluded because they reported taking a nap between the study and test sessions. We analyzed the memory performance for temporally-far non-targets on the subset of participants who reported taking a nap.

For temporally adjacent trials, there were main effects of Trial Type (*F*(1,15)=12.52, *p*=.003, *η_p_²*=.46), such that accuracy for non-targets was better than for control trials, and main effects of Semantic Relatedness (*F*(1,15)=9.57, *p*=.007, *η_p_*²=.39), such that performance for related trials were generally better than for unrelated trials. There was no significant interaction between Trial Type and Relatedness (*F*(1,15)=.88, *p*=.362, *η_p_*²=.056). For temporally far trials, there was a trend level interaction between Trial Type and Relatedness (*F*(1,15)=3.21, *p*=.093, *η_p_*²=.17) and main effect of Relatedness (*F*(1,15)=3.66, *p*=.075, *η_p_*²=.20). There was no significant main effect of Trial Type (*F*(1,15)=2.34, *p*=.15, *η_p_*²=.14).
